# Exposure to Toxicants Affects Everyone, Especially the Very Young

**DOI:** 10.3390/ijms23137232

**Published:** 2022-06-29

**Authors:** Louise C. Abbott

**Affiliations:** Department of Veterinary Integrative Biosciences, College of Veterinary Medicine and Biomedical Sciences, Texas A&M University, College Station, TX 77843-4458, USA; lcabbott02@earthlink.net; Tel.: +1-541-254-0779

Toxicology is an incredibly complex and diverse area of biomedical science that includes numerous areas of specialization. The overarching goals for investigators working in all areas of toxicology are to identify and define the exposures to potential toxicants, assess the risks, and mitigate the impacts. This Special Issue, Reproductive and Developmental Toxicology 2.0, in the *International Journal of Molecular Sciences*, provides up-to-date information about the effects of a range of toxicants on the reproduction and development of many animal species, including humans. As so often is the case, the results from toxicant exposures to animal species can serve as sentinels for human toxicant exposures. Furthermore, reproductive systems, as well as developing embryos and fetuses, display greater risk from exposure to most toxicants, from pharmaceuticals to environmental contaminants. Developing new alternative models, including in vivo, in vitro, or in silico models, is critical to increasing our understanding of how toxicants affect reproduction and development. Not only is modeling critical, but it also is essential to be able to carry out research at all levels of scientific inquiry—from molecules to integrated systems of organisms.

Currently, there are ten articles published in this Special Issue, providing a broad sampling of this vital and vibrant area of toxicology research. Several themes emerge when looking at the articles included in this Special Issue. First, there is a distinct emphasis on developmental toxicology, which indicates its importance in the broader area of toxicology. In the past, the assessment of developmental toxicity has utilized animal studies, primary mammals, the vast majority being rodents. However, new, more advanced, high-throughput modeling systems are rapidly being developed. As evidence of this shift in research focus, six of the ten articles report on studies that utilize non-mammalian testing systems, including *Drosophila melanogaster* [1,2], sea urchin embryos [3], choroid plexus cell culture [4], zebrafish embryos [5], *Caenorhabditis elegans* [2], and cultured human cells [6]. Regulatory agencies have gradually increased their approval rate of these alternatives to whole-animal testing that primarily use mammals [7], albeit slowly. Two additional articles also focused on developmental toxicity [8] and embryotoxicity [9], resulting in 80% of the articles discussing some aspect of developmental or embryotoxicity. Another theme is the importance of exposure to heavy metals [1,4,5,9], aluminum [10], and PAHs and PCBs [3]. Environmental exposures to a wide range of toxic compounds exert critical, adverse influences on reproduction and the developing embryo and fetus. Thus, it is essential to increase our understanding of these impacts to develop new preventions and therapies. A third theme that emerged from this Special Issue focused on the developing nervous system [2,4,9,10], which is highly susceptible to toxicant exposure. While considerable progress has been made in revealing neurotoxicants, numerous challenges remain. Finally, one article in this Special Issue described several experimental models used to study drug transporters in the human placenta [11]. Understanding how drugs are transported across the placenta is essential for maintaining the health of pregnant women and the developing embryos and fetuses.
